# Triaqua­(*N* ^2^,*N* ^4^-di-2-pyridylpyrimidine-2,4-diamine)cobalt(II) fumarate

**DOI:** 10.1107/S1600536808032613

**Published:** 2008-10-15

**Authors:** Ming-hua Yang

**Affiliations:** a Department of Chemistry, Lishui University, Lishui 323000, People’s Republic of China

## Abstract

The Co atom in the title compound, [Co(C_14_H_12_N_6_)(H_2_O)_3_]C_4_H_2_O_4_, has a *mer*-CoN_3_O_3_ octa­hedral coordination arising from the tridentate *N* ^2^,*N* ^4^-di-2-pyridylpyrimidine-2,4-diamine (tpda) ligand and three coordinated water mol­ecules. The asymmetric unit contains two fumarate half-anions, both completed by inversion symmetry. A network of N—H⋯O and O—H⋯O hydrogen bonds leads to a three-dimensional network in the crystal structure.

## Related literature

For a related structure, see: Fang *et al.* (2005 ▶). For background, see: Sheu *et al.* (1996 ▶); Peng *et al.* (2000 ▶).
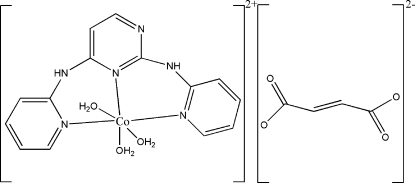


## Experimental

### 

#### Crystal data

[Co(C_14_H_12_N_6_)(H_2_O)_3_]C_4_H_2_O_4_
*M* *_r_* = 491.33
Monoclinic, 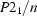

*a* = 9.3239 (3) Å
*b* = 17.1115 (6) Å
*c* = 13.1395 (5) Å
β = 96.224 (1)°
*V* = 2084.00 (13) Å^3^
*Z* = 4
Mo *K*α radiation
μ = 0.88 mm^−1^
*T* = 298 (2) K
0.29 × 0.25 × 0.18 mm

#### Data collection

Bruker APEX CCD diffractometer
Absorption correction: multi-scan (*SADABS*; Sheldrick, 1996 ▶) *T*_min_ = 0.785, *T*_max_ = 0.858
10365 measured reflections
3746 independent reflections
3363 reflections with *I* > 2σ(*I*)
*R*_int_ = 0.014

#### Refinement

*R*[*F* ^2^ > 2σ(*F* ^2^)] = 0.026
*wR*(*F* ^2^) = 0.078
*S* = 0.86
3746 reflections
307 parameters
9 restraints
H atoms treated by a mixture of independent and constrained refinement
Δρ_max_ = 0.28 e Å^−3^
Δρ_min_ = −0.33 e Å^−3^

### 

Data collection: *SMART* (Bruker, 1999 ▶); cell refinement: *SAINT* (Bruker, 1999 ▶); data reduction: *SAINT*; program(s) used to solve structure: *SHELXS97* (Sheldrick, 2008 ▶); program(s) used to refine structure: *SHELXL97* (Sheldrick, 2008 ▶); molecular graphics: *SHELXTL* (Sheldrick, 2008 ▶); software used to prepare material for publication: *SHELXTL*.

## Supplementary Material

Crystal structure: contains datablocks I, global. DOI: 10.1107/S1600536808032613/hb2798sup1.cif

Structure factors: contains datablocks I. DOI: 10.1107/S1600536808032613/hb2798Isup2.hkl

Additional supplementary materials:  crystallographic information; 3D view; checkCIF report

## Figures and Tables

**Table 1:** Selected bond lengths (Å)

Co1—O1	2.0989 (13)
Co1—O2	2.0905 (13)
Co1—O3	2.0396 (13)
Co1—N1	2.0790 (15)
Co1—N3	2.0624 (14)
Co1—N5	2.0803 (16)

**Table 2:** Hydrogen-bond geometry (Å, °)

*D*—H⋯*A*	*D*—H	H⋯*A*	*D*⋯*A*	*D*—H⋯*A*
O2—H2*D*⋯O4^i^	0.836 (10)	1.943 (14)	2.7396 (19)	159 (2)
O2—H2*C*⋯O6^ii^	0.841 (10)	1.898 (10)	2.7374 (18)	176 (3)
O3—H3*C*⋯O7^ii^	0.840 (9)	1.858 (11)	2.6929 (18)	172 (3)
O3—H3*D*⋯O5^iii^	0.832 (10)	1.734 (11)	2.559 (2)	171 (2)
O1—H1*D*⋯O6^iv^	0.829 (10)	1.930 (13)	2.7374 (19)	165 (3)
O1—H1*C*⋯O4^iii^	0.832 (10)	2.001 (13)	2.8143 (19)	166 (2)
N4—H4*A*⋯O5^v^	0.86	1.93	2.782 (2)	169
N2—H2⋯O7^vi^	0.86	2.28	3.002 (2)	141

Symmetry codes: (i) 
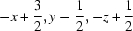
; (ii) 
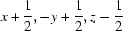
; (iii) 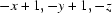; (iv) 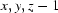; (v) 
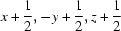
; (vi) 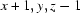.

**Fig. 1.**
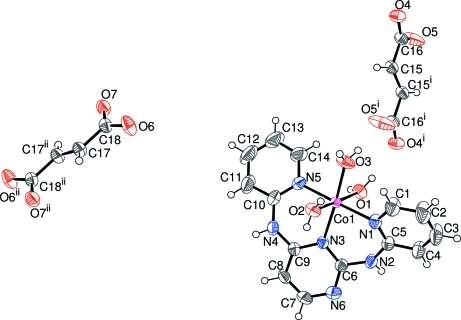
The molecular structure of (I), showing 50% probability displacement ellipsoids for the non-hydrogen atoms. Symmetry codes: (i) 1-x, 1-y, -z; (ii) 1-x, -y, 2-z.

**Fig. 2.**
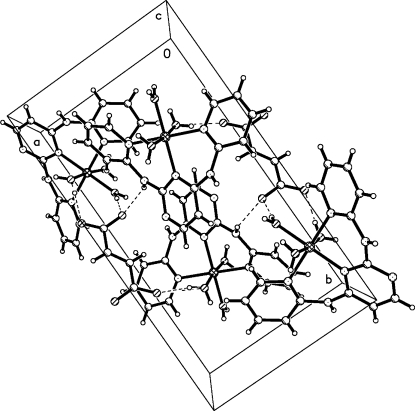
The packing diagram of (I), viewed along the a axis; hydrogen bonds are shown as dashed lines.

## supplementary crystallographic information

### Comment

Transition metal complexes with polypyridylamine ligands, possessing diverse structures and unusual optical and electromagnetic properties (Sheu *et al.*,1996), have aroused great interest among researchers. The tri-pyridyldiamine ligand can exhibit donor as well as acceptor properties and can act as a chelating ligand (Peng *et al.*, 2000). In this paper, we report the synthesis and crystal structure of the title compound, (I), (Fig. 1).

The Co atom in (I) has an octahedral coordination formed by the N,N,N-tridentate tpda ligand and three coordinated water molecules. The tpda ligand is tri-coordinated, with the peripheral N1 and N5 atoms in the axial positions [N1—Co1—N5 = 174.48 (6)°] and the central N3 atom in the equatorial plane of the bipyramid.  The remaining two equatorial positions are occupied by water molecules (Table 1). The three pyridine rings of the tpda ligand are not coplanar: the dihedral angles between the planes of the central pyridine ring and two peripheral rings are 18.5 (4) and 26.4 (2)° respectively.

The H atoms of both NH groups of the tpda ligand and coordinated water molecules are involved in hydrogen bonds with O atoms of carboxylate groups of fumarate which link the complex molecules to form an infinite three-dimensional network (Table 2, Fig. 2).

The molcular configuration of (I) is similar to that of [2,6-bis(2-pyridylamino)pyridine]dinitrato cadmium monohydrate (Fang *et al.*, 2005).

### Experimental

Tpda (0.025 g, 0.07 mmol), Co(NO_3_)2 (0.026 g, 0.1 mmol), fumaric acid (0.023 g, 0.09 mmol) and NaOH (0.041 g, 0.1 mmol) were mixed in acetonitrile, and the mixture was heated for six hours under reflux with stirring. The resultant was then filtered to give a solution which was infiltrated by diethyl ether in a closed vessel, one week later some pink blocks of (I) were obtained.

### Refinement

The water H atoms were located in a difference map and refined with the restraint O—H = 0.85 (1)Å and a fixed U_iso_ value of 0.08Å^2^. The other H atoms were positioned geometrically (C—H = 0.93Å, N—H = 0.86Å) and refined as riding with *U*_iso_(H) = 1.2*U*_eq_(carrier).

### Crystal data

**Table:**

[Co(C_14_H_12_N_6_)(H_2_O)_3_]C_4_H_2_O_4_	*F*(000) = 1012
*M**_r_* = 491.33	*D*_x_ = 1.566 Mg m^−^^3^
Monoclinic, *P*2_1_/*n*	Mo *K*α radiation, λ = 0.71073 Å
Hall symbol: -P 2yn	Cell parameters from 3746 reflections
*a* = 9.3239 (3) Å	θ = 2.0–25.2°
*b* = 17.1115 (6) Å	µ = 0.88 mm^−^^1^
*c* = 13.1395 (5) Å	*T* = 298 K
β = 96.224 (1)°	Block, pink
*V* = 2084.00 (13) Å^3^	0.29 × 0.25 × 0.18 mm
*Z* = 4	

### Data collection

**Table:**

Bruker APEX CCD diffractometer	3746 independent reflections
Radiation source: fine-focus sealed tube	3363 reflections with *I* > 2σ(*I*)
graphite	*R*_int_ = 0.014
ω scans	θ_max_ = 25.2°, θ_min_ = 2.0°
Absorption correction: multi-scan (SADABS; Sheldrick, 1996)	*h* = −11→8
*T*_min_ = 0.785, *T*_max_ = 0.858	*k* = −18→20
10365 measured reflections	*l* = −15→15

### Refinement

**Table:**

Refinement on *F*^2^	Primary atom site location: structure-invariant direct methods
Least-squares matrix: full	Secondary atom site location: difference Fourier map
*R*[*F*^2^ > 2σ(*F*^2^)] = 0.026	Hydrogen site location: inferred from neighbouring sites
*wR*(*F*^2^) = 0.078	H atoms treated by a mixture of independent and constrained refinement
*S* = 0.86	*w* = 1/[σ^2^(*F*_o_^2^) + (0.06*P*)^2^ + 0.982*P*] where *P* = (*F*_o_^2^ + 2*F*_c_^2^)/3
3746 reflections	(Δ/σ)_max_ = 0.001
307 parameters	Δρ_max_ = 0.28 e Å^−^^3^
9 restraints	Δρ_min_ = −0.33 e Å^−^^3^

### Special details

**Table:**

Geometry. All e.s.d.'s (except the e.s.d. in the dihedral angle between two l.s. planes) are estimated using the full covariance matrix. The cell e.s.d.'s are taken into account individually in the estimation of e.s.d.'s in distances, angles and torsion angles; correlations between e.s.d.'s in cell parameters are only used when they are defined by crystal symmetry. An approximate (isotropic) treatment of cell e.s.d.'s is used for estimating e.s.d.'s involving l.s. planes.
Refinement. Refinement of *F*^2^ against ALL reflections. The weighted *R*-factor *wR* and goodness of fit *S* are based on *F*^2^, conventional *R*-factors *R* are based on *F*, with *F* set to zero for negative *F*^2^. The threshold expression of *F*^2^ > σ(*F*^2^) is used only for calculating *R*-factors(gt) *etc*. and is not relevant to the choice of reflections for refinement. *R*-factors based on *F*^2^ are statistically about twice as large as those based on *F*, and *R*- factors based on ALL data will be even larger.

### Fractional atomic coordinates and isotropic or equivalent isotropic displacement parameters (Å^2^)

**Table:**

	*x*	*y*	*z*	*U*_iso_*/*U*_eq_	
Co1	0.89198 (2)	0.212482 (12)	0.174122 (15)	0.02637 (9)	
N1	1.07828 (17)	0.25958 (9)	0.12520 (12)	0.0386 (3)	
N2	1.12613 (18)	0.14671 (9)	0.03083 (12)	0.0428 (4)	
H2	1.1513	0.1318	−0.0272	0.051*	
N3	0.96644 (15)	0.10180 (8)	0.14666 (11)	0.0311 (3)	
N4	0.81107 (18)	0.04503 (9)	0.25721 (12)	0.0418 (4)	
H4A	0.8147	0.0119	0.3066	0.050*	
N5	0.69822 (17)	0.16405 (10)	0.20828 (12)	0.0411 (4)	
N6	1.1419 (2)	0.01872 (10)	0.08085 (15)	0.0527 (4)	
O1	0.80120 (15)	0.22962 (8)	0.02251 (10)	0.0413 (3)	
O2	0.98217 (16)	0.20786 (7)	0.32677 (10)	0.0404 (3)	
O3	0.82120 (18)	0.31928 (8)	0.21618 (11)	0.0515 (4)	
O4	0.31016 (15)	0.62641 (8)	0.05353 (10)	0.0449 (3)	
O5	0.2958 (2)	0.57346 (11)	−0.09942 (12)	0.0843 (7)	
O6	0.56949 (16)	0.15459 (8)	0.92018 (12)	0.0521 (4)	
O7	0.35145 (14)	0.10201 (8)	0.89469 (10)	0.0432 (3)	
C1	1.1139 (3)	0.33447 (13)	0.14869 (18)	0.0572 (6)	
H1A	1.0742	0.3578	0.2031	0.069*	
C2	1.2052 (3)	0.37778 (15)	0.0965 (2)	0.0756 (8)	
H2A	1.2276	0.4291	0.1152	0.091*	
C3	1.2637 (3)	0.34310 (15)	0.0147 (2)	0.0701 (7)	
H3A	1.3229	0.3718	−0.0241	0.084*	
C4	1.2337 (3)	0.26690 (14)	−0.00838 (18)	0.0548 (5)	
H4	1.2728	0.2426	−0.0624	0.066*	
C5	1.1431 (2)	0.22600 (11)	0.05069 (14)	0.0389 (4)	
C6	1.0754 (2)	0.08795 (10)	0.08910 (14)	0.0356 (4)	
C7	1.0969 (2)	−0.04032 (12)	0.13765 (18)	0.0523 (5)	
H7	1.1423	−0.0886	0.1355	0.063*	
C8	0.9887 (2)	−0.03233 (11)	0.19754 (15)	0.0425 (4)	
H8	0.9600	−0.0737	0.2365	0.051*	
C9	0.9231 (2)	0.03917 (10)	0.19853 (13)	0.0348 (4)	
C10	0.6933 (2)	0.09433 (12)	0.25191 (15)	0.0423 (4)	
C11	0.5709 (3)	0.06721 (17)	0.2948 (2)	0.0669 (7)	
H11	0.5709	0.0186	0.3263	0.080*	
C12	0.4505 (3)	0.1148 (2)	0.2887 (3)	0.0845 (9)	
H12	0.3688	0.0992	0.3180	0.101*	
C13	0.4526 (3)	0.1857 (2)	0.2388 (2)	0.0773 (8)	
H13	0.3714	0.2176	0.2317	0.093*	
C14	0.5759 (2)	0.20770 (15)	0.2004 (2)	0.0587 (6)	
H14	0.5766	0.2555	0.1667	0.070*	
C15	0.4580 (2)	0.51840 (11)	0.02931 (13)	0.0376 (4)	
H15	0.4687	0.5068	0.0989	0.045*	
C16	0.3459 (2)	0.57780 (10)	−0.00850 (14)	0.0362 (4)	
C17	0.5353 (2)	0.03277 (11)	0.99689 (14)	0.0372 (4)	
H17	0.6254	0.0371	1.0344	0.045*	
C18	0.4805 (2)	0.10121 (10)	0.93323 (13)	0.0353 (4)	
H1C	0.782 (3)	0.2754 (7)	0.005 (2)	0.080*	
H1D	0.733 (2)	0.2009 (12)	0.001 (2)	0.080*	
H3D	0.785 (3)	0.3513 (12)	0.1732 (13)	0.080*	
H3C	0.827 (3)	0.3404 (14)	0.2742 (9)	0.080*	
H2C	1.007 (3)	0.2514 (8)	0.353 (2)	0.080*	
H2D	1.044 (2)	0.1753 (10)	0.350 (2)	0.080*	

### Atomic displacement parameters (Å^2^)

**Table:**

	*U*^11^	*U*^22^	*U*^33^	*U*^12^	*U*^13^	*U*^23^
Co1	0.02877 (14)	0.02434 (14)	0.02585 (14)	0.00162 (8)	0.00225 (9)	−0.00098 (8)
N1	0.0415 (9)	0.0367 (8)	0.0378 (8)	−0.0084 (7)	0.0053 (7)	−0.0034 (6)
N2	0.0500 (10)	0.0384 (9)	0.0432 (9)	−0.0037 (7)	0.0204 (7)	−0.0032 (7)
N3	0.0336 (8)	0.0285 (7)	0.0315 (7)	−0.0020 (6)	0.0049 (6)	−0.0015 (6)
N4	0.0486 (9)	0.0378 (8)	0.0410 (9)	−0.0051 (7)	0.0139 (7)	0.0064 (7)
N5	0.0344 (8)	0.0476 (9)	0.0419 (9)	0.0019 (7)	0.0063 (7)	−0.0009 (7)
N6	0.0538 (11)	0.0425 (10)	0.0641 (11)	0.0088 (8)	0.0162 (9)	−0.0029 (8)
O1	0.0443 (8)	0.0391 (7)	0.0381 (7)	−0.0007 (6)	−0.0059 (6)	0.0020 (6)
O2	0.0501 (8)	0.0356 (7)	0.0338 (7)	0.0059 (6)	−0.0036 (6)	−0.0028 (5)
O3	0.0763 (10)	0.0389 (8)	0.0367 (7)	0.0209 (7)	−0.0056 (7)	−0.0068 (6)
O4	0.0464 (8)	0.0398 (7)	0.0477 (8)	0.0067 (6)	0.0009 (6)	−0.0092 (6)
O5	0.1314 (17)	0.0680 (11)	0.0454 (9)	0.0625 (12)	−0.0268 (10)	−0.0174 (8)
O6	0.0531 (8)	0.0400 (8)	0.0595 (9)	−0.0146 (7)	−0.0111 (7)	0.0168 (6)
O7	0.0394 (7)	0.0431 (7)	0.0461 (7)	0.0006 (6)	0.0008 (6)	0.0109 (6)
C1	0.0691 (15)	0.0468 (12)	0.0583 (13)	−0.0196 (11)	0.0183 (11)	−0.0138 (10)
C2	0.097 (2)	0.0503 (14)	0.0836 (18)	−0.0367 (14)	0.0304 (16)	−0.0141 (13)
C3	0.0840 (18)	0.0588 (15)	0.0724 (16)	−0.0314 (14)	0.0309 (14)	−0.0004 (12)
C4	0.0608 (14)	0.0522 (12)	0.0550 (13)	−0.0138 (11)	0.0225 (11)	−0.0006 (10)
C5	0.0384 (10)	0.0400 (10)	0.0384 (10)	−0.0056 (8)	0.0051 (8)	0.0000 (8)
C6	0.0361 (9)	0.0350 (9)	0.0362 (9)	−0.0015 (7)	0.0062 (7)	−0.0028 (7)
C7	0.0587 (13)	0.0326 (10)	0.0659 (14)	0.0109 (9)	0.0079 (11)	0.0020 (9)
C8	0.0549 (12)	0.0283 (9)	0.0450 (10)	0.0014 (8)	0.0083 (9)	0.0069 (8)
C9	0.0400 (9)	0.0329 (9)	0.0313 (8)	−0.0043 (7)	0.0034 (7)	−0.0007 (7)
C10	0.0396 (10)	0.0512 (12)	0.0371 (10)	−0.0055 (9)	0.0090 (8)	−0.0025 (8)
C11	0.0558 (14)	0.0774 (17)	0.0714 (15)	−0.0124 (13)	0.0249 (12)	0.0100 (13)
C12	0.0458 (14)	0.122 (3)	0.091 (2)	−0.0025 (16)	0.0318 (14)	0.0096 (19)
C13	0.0408 (13)	0.106 (2)	0.087 (2)	0.0129 (14)	0.0168 (13)	0.0048 (18)
C14	0.0410 (12)	0.0697 (16)	0.0651 (15)	0.0102 (10)	0.0054 (10)	0.0038 (11)
C15	0.0460 (10)	0.0327 (9)	0.0335 (9)	0.0025 (8)	0.0009 (7)	0.0030 (7)
C16	0.0419 (10)	0.0295 (9)	0.0362 (9)	0.0008 (7)	−0.0006 (8)	−0.0026 (7)
C17	0.0382 (10)	0.0358 (9)	0.0364 (9)	−0.0010 (7)	−0.0016 (7)	0.0053 (7)
C18	0.0420 (10)	0.0329 (9)	0.0308 (8)	−0.0017 (8)	0.0027 (7)	0.0020 (7)

### Geometric parameters (Å, °)

**Table:**

Co1—O1	2.0989 (13)	O6—C18	1.258 (2)
Co1—O2	2.0905 (13)	O7—C18	1.254 (2)
Co1—O3	2.0396 (13)	C1—C2	1.369 (3)
Co1—N1	2.0790 (15)	C1—H1A	0.9300
Co1—N3	2.0624 (14)	C2—C3	1.390 (4)
Co1—N5	2.0803 (16)	C2—H2A	0.9300
N1—C5	1.335 (2)	C3—C4	1.361 (3)
N1—C1	1.351 (3)	C3—H3A	0.9300
N2—C6	1.378 (2)	C4—C5	1.396 (3)
N2—C5	1.387 (2)	C4—H4	0.9300
N2—H2	0.8600	C7—C8	1.352 (3)
N3—C6	1.352 (2)	C7—H7	0.9300
N3—C9	1.355 (2)	C8—C9	1.368 (3)
N4—C9	1.368 (2)	C8—H8	0.9300
N4—C10	1.381 (3)	C10—C11	1.405 (3)
N4—H4A	0.8600	C11—C12	1.381 (4)
N5—C10	1.327 (3)	C11—H11	0.9300
N5—C14	1.358 (3)	C12—C13	1.380 (4)
N6—C6	1.347 (2)	C12—H12	0.9300
N6—C7	1.350 (3)	C13—C14	1.358 (4)
O1—H1C	0.832 (10)	C13—H13	0.9300
O1—H1D	0.829 (10)	C14—H14	0.9300
O2—H2C	0.841 (10)	C15—C15^i^	1.317 (4)
O2—H2D	0.836 (10)	C15—C16	1.503 (3)
O3—H3D	0.832 (10)	C15—H15	0.9300
O3—H3C	0.840 (9)	C17—C17^ii^	1.308 (4)
O4—C16	1.235 (2)	C17—C18	1.497 (2)
O5—C16	1.237 (2)	C17—H17	0.9300
			
O3—Co1—N3	174.38 (6)	C4—C3—H3A	120.2
O3—Co1—N1	92.41 (7)	C2—C3—H3A	120.2
N3—Co1—N1	89.63 (6)	C3—C4—C5	118.6 (2)
O3—Co1—N5	89.11 (7)	C3—C4—H4	120.7
N3—Co1—N5	89.35 (6)	C5—C4—H4	120.7
N1—Co1—N5	174.48 (6)	N1—C5—N2	120.50 (17)
O3—Co1—O2	83.24 (5)	N1—C5—C4	122.78 (19)
N3—Co1—O2	91.44 (5)	N2—C5—C4	116.69 (18)
N1—Co1—O2	92.79 (6)	N6—C6—N3	125.42 (17)
N5—Co1—O2	92.66 (6)	N6—C6—N2	114.11 (16)
O3—Co1—O1	91.31 (6)	N3—C6—N2	120.46 (16)
N3—Co1—O1	94.07 (6)	N6—C7—C8	122.84 (19)
N1—Co1—O1	85.23 (6)	N6—C7—H7	118.6
N5—Co1—O1	89.44 (6)	C8—C7—H7	118.6
O2—Co1—O1	174.13 (5)	C7—C8—C9	117.28 (18)
C5—N1—C1	117.16 (17)	C7—C8—H8	121.4
C5—N1—Co1	121.31 (12)	C9—C8—H8	121.4
C1—N1—Co1	119.33 (14)	N3—C9—N4	120.86 (16)
C6—N2—C5	130.39 (16)	N3—C9—C8	123.00 (17)
C6—N2—H2	114.8	N4—C9—C8	116.13 (16)
C5—N2—H2	114.8	N5—C10—N4	120.42 (17)
C6—N3—C9	115.20 (15)	N5—C10—C11	122.6 (2)
C6—N3—Co1	123.13 (11)	N4—C10—C11	117.0 (2)
C9—N3—Co1	120.93 (11)	C12—C11—C10	118.2 (3)
C9—N4—C10	131.96 (16)	C12—C11—H11	120.9
C9—N4—H4A	114.0	C10—C11—H11	120.9
C10—N4—H4A	114.0	C13—C12—C11	119.5 (2)
C10—N5—C14	117.34 (18)	C13—C12—H12	120.3
C10—N5—Co1	121.56 (13)	C11—C12—H12	120.3
C14—N5—Co1	120.41 (15)	C14—C13—C12	118.4 (3)
C6—N6—C7	116.15 (17)	C14—C13—H13	120.8
Co1—O1—H1C	117 (2)	C12—C13—H13	120.8
Co1—O1—H1D	118 (2)	N5—C14—C13	123.9 (2)
H1C—O1—H1D	109.1 (16)	N5—C14—H14	118.1
Co1—O2—H2C	114.8 (18)	C13—C14—H14	118.1
Co1—O2—H2D	124.7 (19)	C15^i^—C15—C16	124.5 (2)
H2C—O2—H2D	106.8 (15)	C15^i^—C15—H15	117.8
Co1—O3—H3D	121.7 (17)	C16—C15—H15	117.8
Co1—O3—H3C	129.8 (17)	O4—C16—O5	125.24 (18)
H3D—O3—H3C	108.4 (15)	O4—C16—C15	117.70 (16)
N1—C1—C2	123.4 (2)	O5—C16—C15	117.05 (16)
N1—C1—H1A	118.3	C17^ii^—C17—C18	124.2 (2)
C2—C1—H1A	118.3	C17^ii^—C17—H17	117.9
C1—C2—C3	118.3 (2)	C18—C17—H17	117.9
C1—C2—H2A	120.8	O7—C18—O6	123.72 (17)
C3—C2—H2A	120.8	O7—C18—C17	119.26 (16)
C4—C3—C2	119.5 (2)	O6—C18—C17	117.01 (16)
			
O3—Co1—N1—C5	−151.20 (15)	C3—C4—C5—N2	−174.2 (2)
N3—Co1—N1—C5	34.03 (15)	C7—N6—C6—N3	−1.9 (3)
O2—Co1—N1—C5	125.45 (15)	C7—N6—C6—N2	179.18 (18)
O1—Co1—N1—C5	−60.08 (15)	C9—N3—C6—N6	−0.5 (3)
O3—Co1—N1—C1	11.43 (17)	Co1—N3—C6—N6	169.80 (15)
N3—Co1—N1—C1	−163.33 (17)	C9—N3—C6—N2	178.32 (16)
O2—Co1—N1—C1	−71.91 (17)	Co1—N3—C6—N2	−11.4 (2)
O1—Co1—N1—C1	102.55 (17)	C5—N2—C6—N6	−144.6 (2)
N1—Co1—N3—C6	−15.88 (14)	C5—N2—C6—N3	36.5 (3)
N5—Co1—N3—C6	158.70 (14)	C6—N6—C7—C8	1.9 (3)
O2—Co1—N3—C6	−108.66 (14)	N6—C7—C8—C9	0.5 (3)
O1—Co1—N3—C6	69.31 (14)	C6—N3—C9—N4	−178.14 (16)
N1—Co1—N3—C9	153.86 (14)	Co1—N3—C9—N4	11.3 (2)
N5—Co1—N3—C9	−31.56 (14)	C6—N3—C9—C8	3.1 (3)
O2—Co1—N3—C9	61.08 (14)	Co1—N3—C9—C8	−167.37 (15)
O1—Co1—N3—C9	−120.95 (13)	C10—N4—C9—N3	26.2 (3)
O3—Co1—N5—C10	−141.78 (15)	C10—N4—C9—C8	−155.0 (2)
N3—Co1—N5—C10	32.82 (15)	C7—C8—C9—N3	−3.2 (3)
O2—Co1—N5—C10	−58.59 (15)	C7—C8—C9—N4	178.03 (18)
O1—Co1—N5—C10	126.90 (15)	C14—N5—C10—N4	176.3 (2)
O3—Co1—N5—C14	28.44 (17)	Co1—N5—C10—N4	−13.2 (2)
N3—Co1—N5—C14	−156.96 (17)	C14—N5—C10—C11	−4.0 (3)
O2—Co1—N5—C14	111.63 (17)	Co1—N5—C10—C11	166.54 (18)
O1—Co1—N5—C14	−62.88 (17)	C9—N4—C10—N5	−25.1 (3)
C5—N1—C1—C2	3.8 (4)	C9—N4—C10—C11	155.2 (2)
Co1—N1—C1—C2	−159.5 (2)	N5—C10—C11—C12	1.5 (4)
N1—C1—C2—C3	0.4 (5)	N4—C10—C11—C12	−178.8 (2)
C1—C2—C3—C4	−2.7 (5)	C10—C11—C12—C13	1.8 (5)
C2—C3—C4—C5	0.7 (4)	C11—C12—C13—C14	−2.5 (5)
C1—N1—C5—N2	171.9 (2)	C10—N5—C14—C13	3.3 (4)
Co1—N1—C5—N2	−25.1 (2)	Co1—N5—C14—C13	−167.3 (2)
C1—N1—C5—C4	−6.0 (3)	C12—C13—C14—N5	−0.1 (5)
Co1—N1—C5—C4	157.03 (17)	C15^i^—C15—C16—O4	−155.6 (2)
C6—N2—C5—N1	−15.7 (3)	C15^i^—C15—C16—O5	25.1 (4)
C6—N2—C5—C4	162.4 (2)	C17^ii^—C17—C18—O7	11.0 (3)
C3—C4—C5—N1	3.8 (4)	C17^ii^—C17—C18—O6	−167.7 (2)

Symmetry codes: (i) −*x*+1, −*y*+1, −*z*; (ii) −*x*+1, −*y*, −*z*+2.

### Hydrogen-bond geometry (Å, °)

**Table:**

*D*—H···*A*	*D*—H	H···*A*	*D*···*A*	*D*—H···*A*
O2—H2D···O4^iii^	0.84 (1)	1.94 (1)	2.7396 (19)	159 (2)
O2—H2C···O6^iv^	0.84 (1)	1.90 (1)	2.7374 (18)	176 (3)
O3—H3C···O7^iv^	0.84 (1)	1.86 (1)	2.6929 (18)	172 (3)
O3—H3D···O5^i^	0.83 (1)	1.73 (1)	2.559 (2)	171 (2)
O1—H1D···O6^v^	0.83 (1)	1.93 (1)	2.7374 (19)	165 (3)
O1—H1C···O4^i^	0.83 (1)	2.00 (1)	2.8143 (19)	166 (2)
N4—H4A···O5^vi^	0.86	1.93	2.782 (2)	169
N2—H2···O7^vii^	0.86	2.28	3.002 (2)	141

Symmetry codes: (iii) −*x*+3/2, *y*−1/2, −*z*+1/2; (iv) *x*+1/2, −*y*+1/2, *z*−1/2; (i) −*x*+1, −*y*+1, −*z*; (v) *x*, *y*, *z*−1; (vi) *x*+1/2, −*y*+1/2, *z*+1/2; (vii) *x*+1, *y*, *z*−1.

## References

[bb1] Bruker (1999). *SMART* and *SAINT* Bruker AXS Inc., Madison, Wisconsin, USA.

[bb2] Fang, X.-N., Li, X.-F. & Zeng, X.-R. (2005). *Acta Cryst.* E**61**, m1123–m1125.

[bb3] Peng, S.-M., Wang, C.-C., Jang, Y.-L., Chen, Y.-H., Li, F.-Y., Mou, C.-Y. & Leung, M.-K. (2000). *J. Magn. Mater.* **209**, 80–83.

[bb4] Sheldrick, G. M. (1996). *SADABS* University of Göttingen, Germany.

[bb5] Sheldrick, G. M. (2008). *Acta Cryst.* A**64**, 112–122. 10.1107/S0108767307043930 18156677

[bb6] Sheu, J. T., Liu, T. W. & Peng, S. M. (1996). *Chem. Commun.* pp. 315–316.

